# (Epistemic) Injustice and Resistance in Canadian Research Ethics Governance

**DOI:** 10.1002/eahr.60004

**Published:** 2025-01-03

**Authors:** Sarah Clairmont, Emily Doerksen, Alize Ece Gunay, Phoebe Friesen

**Affiliations:** ^1^ Researcher and PhD candidate in the Department of Philosophy at McGill University; ^2^ Researcher and recent graduate from the Department of Human Genetics and the Department of Equity, Ethics, and Policy at McGill University; ^3^ Medical student and researcher at the Department of Equity, Ethics, and Policy at McGill University; ^4^ Assistant professor in the Department of Equity, Ethics, and Policy, and in the Department of Social Studies of Medicine, at McGill University

**Keywords:** human subjects research, research ethics governance, research ethics boards (REBs), epistemic injustice, epistemic ignorance, Canada

## Abstract

This article brings a philosophical perspective to bear on issues of research ethics governance as it is practiced and organized in Canada. Insofar as the processes and procedures that constitute research oversight are meant to ensure the ethical conduct of research, they are based on ideas or beliefs about what ethical research entails and about which processes will ensure the ethical conduct of research. These ideas and beliefs make up an epistemic infrastructure underlying Canada's system of research ethics governance, but, we argue, extensive efforts by community members to fill gaps in that system suggest that these ideas may be deficient. Our aim is to make these deficiencies explicit through critical analysis by briefly introducing the philosophical literature on epistemic injustice and ignorance, and by drawing on this literature and empirical evidence to examine how injustice and ignorance show up across three levels of research ethics governance: research ethics boards, regulations, and training. Following this critique, and drawing on insights from the same philosophical tradition, we highlight the work that communities across Canada have done to rewrite and rework how research ethics as a site of epistemic resistance is practiced.

The North American Opioid Medication Initiative (NAOMI) was a randomized control trial that took place from 2005‐2008 in British Columbia, in Vancouver's Downtown Eastside, and focused on testing “whether heroin‐assisted therapy benefits people suffering from chronic opiate addictions.”^1^ During the trial, research participants were given safe access to opioids. One participant said of his experience in the trial, “It was a good thing for me while it lasted,” while another said it gave “a huge break in my life as an addict.”^2^ After 12 months, however, participants were given three months to transition to other available treatments, even though participants were only included in the trial if they had found existing treatments ineffective. Understandably, participants found this transition incredibly difficult. Not only did they lose access to heroin‐assisted treatment, which was effective for many, but several participants felt the study failed to consider or address systemic issues that people suffering from opioid dependencies face, including economic and social marginalization, discrimination, and poverty. As one participant put it: “If you just give me the drug all the time are you improving my life? Well, you're improving my life as far as the drug goes. You're probably taking a lot of the stress out of my life, but are you actually doing these other steps?”^3^

In response to these and other failures, a group of study participants formed the NAOMI Patients Association (NPA) to advocate on behalf of past and future trial participants. In 2013, the NPA published *NAOMI Research Survivors: Experiences and Recommendations*, a report documenting trial experiences and outlining ethical recommendations for future drug maintenance programs.^4^ The Study to Assess Long‐term Opioid Medication Effectiveness (SALOME) was launched shortly after the publication of the report, and took place from 2013‐2015, also in the Downtown Eastside. SALOME studied the effectiveness of six months of heroin‐assisted therapy against six months of Dilaudid to improve the health of long‐term opiate users. As was the case with NAOMI, participants were offered only conventional care once the study ended, including methadone maintenance and drug‐free treatment options, even though these treatments were known to be ineffective for SALOME participants.^5^ Following SALOME, the NPA reorganized as the SALOME/NAOMI Association of Patients (SNAP). SNAP continues to offer support, education, and advocacy for people who use drugs, in Canada and internationally.^6^

The experiences of members of the NPA and, later, SNAP raise questions about the processes by which research is determined to be “ethical” in Canada. The rationale behind research ethics as a process and a discipline is often framed in the language of “protection” as systems of research ethics governance emerged in the latter half of the twentieth century in response to various instances of abuse and exploitation within research. For example, children with developmental disabilities were intentionally infected with hepatitis at the U.S.‐based Willowbrook State Hospital; impoverished Black men with syphilis enrolled in the observational U.S. Public Health Service Untreated Syphilis Study at Tuskegee were never informed when a treatment was available; and imprisoned people were given psychedelic drugs without consent as part of the CIA‐funded project MK‐Ultra.^7^ These abuses led to the development of codes of ethics, regulatory bodies, and research review processes, all of which have become integral parts of the research enterprise in Canada and internationally. However, despite the development of various levels of protections for research participants, there are many other community members or groups, beyond the NPA and SNAP, who do not feel represented within or protected by the existing structures of research ethics governance in Canada.^8^

For decades, Indigenous communities have expressed concerns related to exploitative and unethical research practices in which researchers fail to adequately consent participants, disrespect traditional knowledge, fail to share results, and publish stigmatizing narratives.^9^ In response, many have developed strategies, such as community‐based ethics review processes and codes of ethics, to prevent harmful research from taking place and to ensure community benefit.^10^ More recently, other communities have stepped forward, seeking a voice in research ethics governance. Residents in Vancouver's Downtown Eastside published *Research 101: A Manifesto for Ethical Research in the Downtown Eastside*, and community members in the Jane and Finch area, a

**Via the lens of social epistemology, we explore what might underlie the disconnect between a system tasked with ensuring the ethical conduct of research and the many efforts to ensure the ethical conduct of research in communities on the part of those outside that system**.

densely populated low‐income neighborhood in Toronto, in response to a history of extractive, misrepresentative, objectifying, and deficit‐based research, developed the *Principles for Conducting Research in the Jane Finch Community*. Each offers guidance on how researchers interested in conducting research in their communities might better accommodate community needs and prevent unnecessary harm.^11^ These efforts, among other community‐led developments in the space of research oversight, suggest that even though there has been substantial development with regard to the regulation of research in the past several decades, the system of governance is still falling short.

In this article, via the lens of social epistemology, we will explore what might underlie the disconnect between a system tasked with ensuring the ethical conduct of research and the many efforts to ensure the ethical conduct of research in their communities on the part of those outside that system. We focus specifically on processes of research ethics review and consider how they can and do facilitate epistemic injustice and ignorance. First, we briefly introduce the philosophical literature on epistemic injustice and ignorance. Next, we draw on this literature and empirical evidence to examine how injustice and ignorance show up across three levels of research ethics governance: research ethics boards (REBs), regulations, and training. Following this critique, we highlight as sites of epistemic resistance the work that communities across Canada have done to rewrite and rework how research ethics is practiced.

## (EPISTEMIC) INJUSTICE AND IGNORANCE

The term epistemic injustice, coined by Fricker, refers to “a wrong done to someone specifically in their capacity as a knower.”^12^ Fricker distinguishes two broad types of epistemic injustice: testimonial and hermeneutical injustice. Testimonial injustice occurs when a “credibility deficit” is wrongly and unfairly attributed to an individual or group based on prejudice (e.g., when a woman's testimony is dismissed as being “too emotional,” as a result of implicit or explicit bias). Hermeneutical injustice occurs when some significant aspect of a person's or group's experience is obscured from individual and collective understanding because of a more structural prejudice (e.g., the relative absence of queer representation and experiences in children's stories).

Epistemic injustice is comparable to the notion of social exclusion developed in the social sciences to explain how certain groups are prevented from participating in the normal social, cultural, and economic activities of a given society.^13^ Like social exclusion, epistemic injustice concerns both process and output. And just as the harms of social exclusion are not merely social, but include, for example, economic and health harms, the harms of epistemic injustice are not confined to the domain of knowledge. When the relevant input of some individual or group is obstructed, either by interpersonal or structural forces, the consequences can, and often do, constitute other types of harm as well.

Good health care, for instance, depends on expert knowledge from both health care providers and patients. However, health care systems are often structured in a way that systematically and disproportionately renders patients vulnerable to the harms of epistemic injustice: “Pressures of time, the high stakes and burden of responsibility, plus the need for technical or otherwise professional language all conspire to make it all too easy for a doctor to either fail to solicit her patient's relevant epistemic input” or give “short shrift to legitimate questions and concerns.”^14^

Both the doctor and the patient, in this case, are epistemically disadvantaged by the social conditions under which knowledge of the patient's health is sought; the doctor may fail to elicit information needed to treat the patient and the patient may not receive the care she needs. These structural features can compound with prejudice against members of certain social groups and result in discriminatory practices, such as racialized patients receiving inadequate pain treatment or the dismissal of legitimate symptoms of people affected by obesity.^15^

Fricker is not the first to theorize the epistemic dimensions of unjust social arrangements, but her work has played an important role in uniting a subset of literature within the broader tradition of social epistemology (the study of how knowledge is acquired, stored, shared, evaluated, and applied within various social contexts) that has focused specifically on the intersections of knowledge and power.^16^ On these more critical accounts, the social manner in which knowledge is built and operates on a large scale is decidedly nonideal. Human practices of knowledge creation and consumption are riddled with challenges that stem from deeply rooted social disparities and biases, and the result is a knowledge system that tends to favor the narratives or perspectives of a privileged few, while silencing others. This imbalance not only perpetuates existing power structures; it perpetuates *ignorance*.

Ignorance, as it is theorized in relation to epistemic injustice, is not merely about gaps in knowledge; it is a substantive epistemic practice that consists in ongoing efforts to resist knowledge, and ways of knowing, that threaten unjust systems of social organization (hierarchies of race and gender, for example), as well as the unfair advantages that these systems confer onto privileged individuals and groups. Sometimes, these efforts are conscious, even legally sanctioned; sometimes they are unintentional, unconsciously generated, or only tacitly supported. To map an *epistemology of ignorance* is to examine and uncover the specific mechanisms and structures through which epistemic deficits, patterns of ignorance, exclusivity, and insensitivity are created and sustained within a particular context.^17^

In what follows, we map an epistemology of ignorance and injustice across three (interrelated) levels of research ethics governance in Canada: REBs, research ethics regulations, and research ethics training.

## REBS: FAILURES OF REPRESENTATION AND UPTAKE

REBs play a fundamental gatekeeping role in research oversight in Canada. Their primary task is to determine the ethical acceptability of a research proposal. All research involving human participants must obtain approval from an REB before the research project can begin. When we look closely at the functioning of REBs, however, at least two issues arise that systematically disadvantage them as collective epistemic agents. First, REBs are limited in their representation, especially when it comes to local or community expertise. Second, even when REBs are representative of communities, they are at risk of not effectively soliciting and taking up valuable community knowledge. These disadvantages, we argue, not only undermine the epistemic authority granted to the REB but culminate in significant epistemic injustices against both research participants and the REB members who represent them.

### Failures of representation

Feminist standpoint theory holds that experiences of marginalization can sometimes contribute to an epistemic advantage in relation to particular knowledge projects. This view is based on a descriptive claim (*the situated knowledge thesis*) and a normative claim (*the standpoint thesis*). The situated knowledge thesis holds that what one can know is restricted by how one is situated. What individuals come to know is shaped by their social location in a hierarchically structured system of power relations, by the material conditions of their lives, and by the conceptual resources available to them. Whereas the situated knowledge thesis applies equally to all knowers, the standpoint thesis applies to those who have engaged in critical reflection regarding how knowledge is produced in a given domain. As a result, they can develop an epistemic advantage, or standpoint, in relation to relevant knowledge projects. For example, through developing a standpoint, a woman with lived experience of gender‐based discrimination when seeking health care may develop critical insights into how medical knowledge and practices often neglect or pathologize women's health concerns.^18^

The Canadian Tri‐Council Agencies require at least one REB community member, who cannot be affiliated with the REB's institution, to constitute a quorum in the review of studies to “help broaden the perspective and base value of the REB” and to “advance dialogue with and accountability to relevant communities.”^19^ While this requirement includes an acknowledgment of the need for community expertise in ethics review, indicating an appreciation for feminist standpoint theory, empirical evidence suggests that, in practice, REB membership is rarely representative of the research participants these boards are meant to protect. Community members on REBs are often White, older, female, and highly educated (e.g., retired doctors, researchers, lawyers, and others known to those who serve on the REB).^20^ As a result, REBs are unlikely to represent the standpoints of relevant community members, missing out on epistemic contributions that could help them to better understand how risks and benefits might be understood by a given community.^21^ As such, it is unsurprising that research protocols are often approved that exclude populations likely to be impacted by the research (e.g., those who do not speak English), that fail to directly benefit those participating in the research (e.g., by asking questions that are not important to the community), or that utilize outcome measures that are not reflective of participant goals (e.g., mere symptom reduction over quality of life).^22^

There are a variety of reasons for this lack of representation. Crucially, REB membership is burdensome, requiring plenty of free time to prepare for and participate in meetings. Being on an REB requires a substantial amount of reading, familiarity with technical language, such as the kind used in research protocols or policies, and, above all, an interest in participating in this bureaucratic process. Protocols are often hundreds of pages, and to engage in a discussion of the ethicality of the proposed projects members must review and familiarize themselves with several such protocols before each meeting (usually held biweekly or monthly, depending on how busy the REB is). Incentive structures behind REB membership look different for community members as well. For members affiliated with the REB's institution, ethics review can aid in professional development, strengthen a curriculum vitae, affect tenure, and offset duties like teaching or administrative support, but little is offered to motivate community members, who generally volunteer their time without necessarily reaping these rewards. It is also worth considering the ways in which community members—especially those who belong to communities that have been exposed to research harms, exploitation, and over‐research—may be disincentivized to participate in a system that has historically mistreated them.

Another factor impacting REB representation relates to the norms that govern the processes of recruiting individual REB members. Evidence on REB recruitment in Canada is limited, but data from the U.S. suggests that community members serving on REBs tend to be recruited largely through convenience or social connections.^23^ Slaven, a community representative who served on the REB at Montreal's Jewish General Hospital, confirms that this was her experience as well—a friend and fellow REB member initially encouraged her to participate in the review process.^24^

In his discussion of elite capture and epistemic deference, Táíwò is critical of the prevailing norms that convert standpoint theory into practice. As he puts it: “The common approaches to putting these abstract ideas into practice emphasize deference to others [namely, those thought to have attained a standpoint] in conversational contexts, in an effort to fix the distribution of attention: they ask that we pass the mic, believe marginalized people, and give offerings.”^25^ And while the motivations behind such practices are admirable, structures of injustice often determine who has epistemic authority, especially in “rooms” of outsized power (boardrooms, classrooms, political parties, and so on). In other words, practices of deference usually mean “handing conversational authority and attentional goods to whoever is already in the room and appears to fit a category associated with oppression—regardless of what they have or have not actually experienced, or what they do or do not actually know about the matter at hand.”^26^ In the case of research oversight, the requirement of a community representative on an REB is intended to acknowledge the importance of the standpoints that community members can bring to research ethics. And yet, due to the burdensome, inaccessible, and often unpublicized nature of REB membership, those often chosen to represent communities may be in these positions “precisely because of the ways in which they are systematically *different from* (and thus potentially unrepresentative of) the very people they are then asked to represent.”^27^

### Failures of uptake

In addition to poor representation of community members within REBs, inequitable distributions of epistemic authority and credibility between individual REB members may prevent the uptake of community voices even when they are present. Indeed, some evidence suggests that the conditions under which ethics review takes place are often such that community knowledge fails to be solicited. As Slaven describes her experience: “As the meetings procedures and the medical terms, abbreviations, acronyms, and other jargon grew more familiar, I began to recognize the specific areas of expertise that each person represented … [A]s the months went by and I became more comfortable, I asked fewer questions—at least fewer basic questions, fewer whys and hows. I think that I began to identify with the professionals—to feel that I was part of the group. And since the group was made up of intelligent, hard‐working, well‐intentioned people who were trying to do good things, I thought: Who am I to question their judgment? Perhaps I became less effective as a result. Perhaps I lost my outsider's perspective—the different point of view I brought to the table. Perhaps I forgot to see myself as the potential research participant, who might very well be ill, frightened, worried, vulnerable, disoriented, too trusting or too tired to ask questions.”^28^

Unfortunately, the kind of self‐silencing Slaven is describing is not uncommon in ethics review. Both community and noncommunity REB members have reported power imbalances that leave community representatives in particular feeling unable to speak out.^29^ In the end, Slaven decided that her expertise was only welcome in limited areas of the review, concluding: “the committee was obviously not counting on my contribution to the discussion of the scientific or medical aspects of the research, so my efforts would best be spent on the informed consent document.”^30^ Slaven's experience is reflected in empirical research on institutional review boards (the equivalent of REBs in the U.S.), in which community members often report that examining consent forms is their only, or primary, task on the committee.^31^ Likewise, when these members were interviewed about why they were recruited, they “immediately began speaking about their lack of expertise in science or knowledge of the institution or ‘playing the ignorant person.’”^32^

To understand how community representatives may be systematically silenced in ethics review it is helpful to introduce the concepts of *testimonial quieting* and *testimonial smothering*, developed by Dotson, a philosopher.^33^ Building on the work of Spivak and Hornsby, Dotson elucidates the audience's role in linguistic communication and the successful transfer of knowledge.^34^ Testimonial quieting, she argues, “occurs when an audience fails to identify a speaker as a knower” as a result of individual or structural prejudice.^35^ To restrict community expertise to consent forms is to devalue or discredit community representatives as knowers. It reinforces elitist assumptions and the belief that community representatives, by virtue of representing their communities, “bring a perspective that is not medical, not scientific, and not academic.”^36^ These assumptions may be related to a distinction that often circulates within research review processes between ethical and scientific aspects of the review process. Many REBs require scientific review of a proposal by peers before ethical review takes place. While it is easy to see how those working in the same area of research (e.g., clinical trials in cardiology) will be more knowledgeable in terms of the science behind a proposal (e.g., is this research asking a novel and worthwhile question within the field? Are the latest findings being taken into account?), this doesn't mean that the scientific questions should be set aside within the ethical review. Indeed, the exclusion of women from clinical trials was long thought to be scientifically appropriate and is now seen as both ethically and scientifically problematic.^37^

The closely related phenomenon of testimonial smothering refers to the “truncating of one's own testimony” and “occurs when a speaker perceives one's immediate audience as unwilling or unable to gain the *appropriate* uptake of proffered testimony.”^38^ Unlike testimonial quieting, testimonial smothering is an act of self‐silencing, though it is no less coerced. Slaven's description of her diminishing involvement in the review process, her habitual deference to or identification with the “experts” in the room, serves as a compelling illustration of testimonial smothering and how the social conditions under which ethics review takes place can function to suppress community knowledge rather than procure it.

Both testimonial quieting and testimonial smothering are socio‐epistemic practices that culminate in what Dotson calls *epistemic violence*, a kind of epistemic injustice that arises when an audience fails “to communicatively reciprocate, either intentionally or unintentionally, in linguistic exchanges owing to pernicious ignorance.”^39^ Such epistemic violence serves to reinforce prejudice against those who are routinely silenced through testimonial quieting and testimonial smothering, depriving many of receiving or contributing relevant testimony, and maintaining a problematic epistemic hierarchy. In what follows, we explain how such ignorance is buttressed in ethics review by the regulations that guide REB decision‐making.

## THE TCPS: A TOOL OF UNIVERSALISM, INDIVIDUALISM, AND COLONIALISM

The primary regulatory document related to research oversight in Canada is the *Tri‐Council Policy Statement: Ethical Conduct for Research Involving Humans*, currently in its second edition, and colloquially known as the TCPS. The TCPS details the responsibilities of researchers, research institutions, and REBs related to research oversight, including the ethical standards that guide REB decision‐making.^40^ All researchers and REB members in Canada are expected to understand the ethical standards outlined in this policy and how to apply them.^41^ It is drafted by the Panel on Research Ethics, a national committee tasked with developing and updating these federal standards, and includes 13 chapters covering a range of topics from the consent process to ethical considerations for research involving human biological materials. Here we focus on how the document functions as a mechanism of ignorance in ethics review because of embedded, implicit commitments to universalism, individualism, and White‐settler colonialism.

### Universalism

The core value of the ethical framework in the TCPS is “respect for human dignity” and the policy's commitment to that value is expressed through its three core principles: respect for persons, concern for welfare, and justice.^42^ Such a framework is, first and foremost, a universal, principlist one. While principlism is regarded as the primary moral theoretical approach to bioethics and research ethics in the Global North, and increasingly around the world, it has been widely criticized for failing to account for non‐Western ways of engaging with, and conceiving of, moral analysis.^43^ As Stevenson and colleagues have written, “the current scope of Western research ethics is not always sufficient for engaging in research with culturally diverse, or non‐Western populations.”^44^ Crucially, by presenting an ethical framework that is meant to apply to all research, regardless of where it takes place, what it consists of, and who it involves, the TCPS suggests that morality in research ethics is universal. As such, local and contextual ethical considerations are likely to remain invisible.^45^

While countless communities across the country are advocating for local review and the importance of community perspectives in guiding research governance, the TCPS leaves little space for such voices, contributing to epistemic exclusions reinforcing ignorance. REB members and researchers familiar with the regulations may be led to believe that they are aware of all relevant ethical considerations, when in fact, they may remain ignorant of considerations relevant to a particular community. For example, when working with a particular community, they may fail to consider whether the way they describe a patient group in a manuscript may be taken as offensive and stigmatizing by that group (e.g., using the term schizophrenic as a noun), even though it may be a description used in the academic literature.

### Individualism

In taking up respect for human dignity and respect for persons as guiding principles, the TCPS offers an epistemic and ethical framework that is not only universalist, but also highly individualistic. This focus on individuals, as opposed to communities or populations, is borne out in data related to REB processes and decision‐making. Review forms, for example, are often developed by REBs to guide the review process and ensure all ethical aspects of a proposal are considered (e.g., confidentiality, vulnerability, and so on). However, these forms tend to reflect the limited and individualistic focus of the TCPS, to the exclusion of other morally relevant considerations. After assessing review forms from 30 REBs from Canada and the U.S., Flicker et al. found that none of the forms asked about community involvement in identifying the rationale for the research project, and only 13% asked about risks and benefits at a community or societal level.^46^ Similarly, in a Canada‐wide assessment of 89 REB ethics review protocol forms for community‐based participatory research projects, only 20% asked about risks and benefits to communities, while all asked about risks to individuals. All but a single form asked explicitly about individual informed consent, but only 25% of forms asked about community consent.^47^

Such an individualistic focus can contribute to a kind of tunnel vision in REB members, in which they fail to see community‐level concerns or harms when assessing research protocols. As such, REBs “overwhelmingly operate within a traditional framework focused on assessing risk to individuals and not communities.”^48^ This both produces and reinforces ignorance when it comes to risks or benefits of research that may apply at the level of communities. Researchers and REB members may learn to paint a picture of ethical considerations relevant to a given protocol that focuses exclusively on risks and benefits to individual research participants (e.g., risks arising from exposure to an untested medication, benefits from additional care and attention), while failing to see such risks and benefits that arise in communities involved in the project (e.g., risks related to excluded populations like older people or those with child‐bearing potential, benefits in terms of developing a community‐specific intervention). Reinforcing this, Cook and colleagues conducted interviews with Canadian REB members about clinical trial benefits, and found that several participants explained that they had never considered benefits for community members in their role as an REB member.^49^

### Colonialism

The only exception to a presumed universalism in the TCPS is chapter 9, focused on research involving the First Nations, Inuit, and Métis peoples of Canada. The first version of this chapter was published in 1998, but many pointed out that there was a lack of formal consultation with Indigenous communities during the development of these guidelines.^50^ Soon after this criticism, the Canadian Institutes of Health Research (CIHR) undertook extensive consultation with Indigenous communities, organizations, and individuals, as well as researchers, and produced the *CIHR Guidelines for Health Research Involving Aboriginal People* in 2007.^51^ These guidelines were updated in 2010, in collaboration with the other research councils, to form chapter 9 of the TCPS (and have been updated every 4 years since). The chapter describes how researchers and REB members may interpret each of the three core principles in the context of research with Indigenous communities and lists 22 articles describing further requirements. These requirements focus on community engagement, understanding local authority structures, governing bodies, and knowledge keepers, as well as formal research agreements that must be signed in advance of recruitment that delineate plans for the dissemination of results and data stewardship.^52^

While chapter 9 is a laudable development in Canada's system of research oversight, it is still insufficient in practice, upholding and reinforcing structures of White settler colonialism, which has fundamental roots in ignorance.^53^ The chapter leaves significant gaps in REB members’ understanding of the sovereignty, processes, and jurisdiction of Indigenous communities. While the chapter stresses the importance of researchers being aware of local governance, in practice, many “REBs are not sufficiently aware of jurisdictional considerations required in Indigenous research, and even for those who may be, it is not clear to REBs which Indigenous community or body has jurisdiction or governance authority over research, and how to ensure their engagement and consent has been obtained by researchers.”^54^ When interviewed, REB members have acknowledged that they lack a clear understanding of how an overseeing body, like the Mi'kmaw Ethics Watch or community‐based REBs, actually fit into the review process.^55^ In other words, REB members familiar with the TCPS appear to know that local authority structures have an important role to play in determining the ethical acceptability of a research protocol, but they are unclear about what that role is as well as how to ensure it is respected.^56^ As a result of these gaps in knowledge, community review may simply be bypassed, or local codes of ethics may not be considered by the REB. This serves to maintain White settler colonialism, as legitimate means of enacting Indigenous self‐determination fail to be respected during the research review process.

Furthermore, even if cases in which local research authorities are recognized as having the right to review a protocol and sign a research agreement, they often end up with less power than institutional REBs, especially in cases of conflicting views. As Stiegman and Castleden describe, the TCPS “does not give any guidelines to REBs or researchers in terms of how to navigate the tensions that arise when the ethical guidance of Indigenous peoples contradicts that of a university REB.”^57^ As a result, researchers and REB members may end up deferring to the institution that employs them, controls their funding, and grants their tenure and promotion.

**By taking a closer look at how communities have resisted the status quo when it comes to the production of knowledge in research ethics, we can better understand where else expertise is located, and how the current system might respond to such expertise in an epistemically virtuous way**.

As one researcher described it, “It felt as though I was being left with a process where the community had a leash that was two inches long, and every decision that they wanted to make of any significance, [the university REB] had to okay before they could go ahead.”^58^ Given these circumstances, critics have described the Indigenous jurisdiction that the TCPS acknowledges “to be token at best; the colonial academy, represented by the REB, retains ultimate decision‐making over the research process.”^59^

Chapter 9 of the TCPS also promotes community engagement as an “unquestionable good,” while in practice, it is not so simple; “engagement sometimes intersects with existing research burden and fatigue in Indigenous communities,” particularly when it involves unpaid labor on the part of community members.^60^ When one considers the material realities and research burden of under‐resourced communities, engagement is not as simple a recommendation as it is often made out to be. Given this, local research authorities must have the power to resist a textbook ethical project (one that meets all formal requirements) to exercise true self‐determination.^61^

By failing to adequately share power, acknowledge local authorities, and recognize the difficulties and burdens associated with community engagement, chapter 9 promotes a view of research with Indigenous communities as “TCPS plus”—the same principles plus a few more boxes to tick. Such a view may be leading to a reality in which REBs remain insensitive to the complexities and unique considerations related to research in a particular community and ill‐equipped to manage conflicts that might arise between local and institutional governing bodies. As such, communities are denied the opportunity to fairly contribute to the epistemic terrain surrounding research ethics in their community, and research ethics review enacts a familiar colonial dynamic.

## CORE TRAINING: AN INACCESSIBLE RESOURCE

A final level in Canada's system of research oversight is training. All Canadian researchers and REB members are required to complete the Course On Research Ethics (CORE) in order to conduct or review research.^62^ The course introduces researchers and REB members to the TCPS regulations in Canada. While some institutions may require the CORE training be completed only once, others require a recent certificate of completion. Because the CORE training modules are premised upon the ethical framework developed in the TCPS, many of the issues discussed in the previous section apply to ethics training as well. Still, it is worth flagging additional ways the CORE training requirements serve to produce epistemic injustice and reinforce ignorance. For one, the CORE's online format acts as a technological barrier. Many northern communities in Canada, such as the Inuit Nunangat, for example, do not have access to the kind of reliable broadband internet required to complete this training program.^63^

Other examples of the general inaccessibility of the CORE training program come from a participatory action research project called the Overdose Prevention Peer Research Assistant (OPPRA) project.^64^ The project was collaboratively led by academic researchers and representatives from the Overdose Prevention Society, and involved peer research assistants, who were frontline harm reduction workers and/or people who use(d) drugs, as peer research assistants. It was quickly apparent that existing training materials were not ideal for the project at hand. When newly hired participatory research assistants attempted to access the CORE training online, they reported significant challenges. For instance, some of the research assistants did not have computer access outside of the public library or University of British Columbia (UBC) Learning Exchange. Given that the CORE takes several hours to complete, problems of access added a substantial burden for these research assistants. Additionally, for those without an academic background, “the language and jargon used in the modules” was inaccessible.^65^

These details of accessibility may seem small, focusing on jargon, access to computers, or internet infrastructure, but they act as further constraints when it comes to who shapes knowledge. Access issues related to the CORE training may limit who becomes a researcher and is able to contribute to knowledge production, as well as who becomes an REB member, and contributes to overseeing knowledge production. As such, these factors compound injustice and ignorance, limiting whose epistemic resources are taken up in practice.

While we may have painted a pessimistic picture about the state of injustice and the circulation of ignorance within Canada's system of research oversight, there is more to the story than this. Countless expressions and forms of resistance have been developed by communities and in corners of this system. Of particular interest to us are cases of epistemic resistance: community‐led developments that work to counteract the sort of ignorance and injustices documented above. By taking a closer look at how communities across Canada have resisted the status quo when it comes to the production and recognition of knowledge in research ethics, we can better understand where else expertise is located, and how the current system might respond to such expertise in an epistemically virtuous way.

## BEYOND EXCLUSION: RESISTANCE IN COMMUNITY‐LED RESEARCH ETHICS GOVERNANCE

The term *epistemic resistance*, coined by Medina, refers broadly to friction generated through differences in individual and group experiences and expertise that arise in situations of oppression. As epistemic agents, people can—for better or worse—resist our individual expertise and experiences as well as the experiences and expertise of others. Our above analysis demonstrated how valuable community knowledge is problematically resisted throughout Canada's system of research oversight. But, as Medina makes clear, epistemic resistance can also be used to respond to such injustices, considering “an opposing internal force that counteracts epistemic influences can be positive insofar as it is critical, unmasks prejudices and biases, reacts to bodies of ignorance, and so on.”^66^ As such, epistemic resistance can create “beneficial epistemic friction,” helping one to see what was previously unseen and recognize how ignorance is operating in a given domain of knowledge. As a means of responding to ignorance and injustice, Medina describes how resistance requires the use of “epistemic resources and abilities to undermine and change oppressive normative structures and the complacent cognitive‐affective functioning that sustains those structures.”^67^ In the realm of research governance, where ignorance and injustice circulate, such epistemic resistance is a welcome, and sorely needed, corrective. Fortunately, there is no shortage of examples of this kind of resistance in research ethics, and they cut across each level of research governance discussed above.

### REBs

As mentioned, REBs serve as central gatekeepers in relation to knowledge production and maintenance. These knowledge processes are not accessible to many who represent communities impacted by research, nor are they always supportive of, or welcoming to, community member contributions. This limits the expertise on REBs and contributes to ignorance when it comes to community concerns. In response, many communities have developed their own review committees, composed of community members impacted by research who can better represent the views, concerns, and goals of those likely to be research participants.

One longstanding and impactful example can be found in the Kanien'kehá:ka (Mohawk) community of Kahnawá:ke (pronounced gahna'wa:ge), Quebec, in which all research activities associated with the Kahnawake Schools Diabetes Prevention Project (KSDPP) are reviewed for approval by the KSDPP Community Advisory Board (CAB). This independent CAB was developed in recognition of the fact that the institutional REBs at nearby universities were not equipped to represent the views of community members. Because they rarely included members of the Kahnawá:ke community, they were ignorant of Kanien'kehá:ka traditions, culture, and values and how these might impact the evaluation of a research proposal. The KSDPP CAB is made up of volunteers from community organizations and services, community‐based researchers, and the general community.^68^ Each CAB member is recognized for their unique knowledge, experience, and capabilities, and each reviews and approves research projects based on a set of principles that uphold Kanien'kehá:ka traditions, culture, and values.

Prior to formal ethical review by the CAB, researchers are required to present their research proposals to the KSDPP research team for scientific review by the KSDPP scientific director and KSDPP research supervisors. The KSDPP research team reviews protocols for scientific integrity and ethical considerations, such as individual and community level risks, benefits to the community, potential for community capacity‐building, and research data governance. And while the KSDPP research team can work with researchers and give feedback on such scientific and ethical considerations, the CAB holds the final decision‐making power to definitively approve or reject outside research proposals. The CAB also supervises all stages of a health intervention or research project, from design to assessment, ensuring that they are accountable to the community. This structure of ethics review recognizes the CAB as an autonomous ethical authority based on a wide range of community expertise, and outside research partners with the KSDPP hold a shared understanding that community expertise and involvement in decision‐making strengthens research projects and produces outcomes that are beneficial to the community.^69^ This system of oversight resists the federal, established processes of institutional REBs reviewing and approving research according to its alignment with the TCPS; instead, it injects local values into the review process as well, ensuring that community members in Kahnawá:ke will not be exposed to undue risk and will benefit from research taking place in their community and on their lands.

Many other examples of community‐led REBs can be found in Indigenous communities across the country. These include the Manitoulin Anishinaabek Research Review Committee which reviews research taking place on Manitoulin Island in Ontario, and the Mi'kmaw Ethics Watch, which reviews all research “involving collective Mi'kmaw knowledge, culture, arts, spirituality, or traditions, or having the potential to impact treaty or Aboriginal rights.”^70^ These REBs are recognized in the TCPS, which specifies that research ethics review should be sought by the institutional REB and “any responsible community body recognized by the First Nations, Inuit or Metis authority” in advance of recruitment.^71^ However, as mentioned above, REB members are not sufficiently aware of the existence of these alternative review processes or how they fit into the formal review process. As a result, researchers are not always alerted to their obligation to seek community approval before research begins.

Non‐Indigenous communities, having drawn inspiration from such models formalized by Indigenous communities, are also in the process of developing community‐led REBs, but lack recognition accorded by the TCPS. These include members of the Community Research Ethics Workshop from Vancouver's Downtown Eastside, as well as members of the Jane Finch Community Research Partnership. Across the border in the U.S., tribal IRBs have proliferated in recent years, and other community‐led REBs (e.g., in the Bronx, New York and in Flint, Michigan) have also popped up.^72^ While lacking a claim to sovereignty, these communities have also been exposed to harms from exploitative research and over‐research, raising questions about which communities deserve recognition and authority when it comes to community‐level research governance, and why.

### Regulations

Regulations make up the federally recognized body of knowledge when it comes to what counts as ethical research, but they fail to include ethical considerations that may be meaningful and important to specific communities. In response to these omissions, several communities in Canada have developed, and continue to develop, local guidelines, codes of ethics, manifestos, and protocols. These guidelines aim to educate researchers about the unique histories, values, and concerns of communities that are not represented, or included, in the TCPS. Because the TCPS serves to paint a universal picture of what ethical research ought to look like, local considerations are often excluded, reinforcing ignorance in researchers and REB members who may consult the regulations in search of the one right answer to an ethical dilemma. Many community‐developed guidelines disrupt this way of thinking, offering instead a highly localized picture of what matters.

One example can be found in the *Guidelines for Research with Aboriginal Women*, published by Quebec Native Women Inc., also known as Femmes Autochtones du Québec. Developed with the support of Basile, a researcher originally from the Atikamekw community of Wemotaci, the Quebec Native Women guidelines aim to help Indigenous women, decision‐makers within Indigenous communities, and researchers “manage the many research proposals received and make informed decisions on whether or not to become involved in those projects.”^73^ They include tools and methods for researchers, such as culturally relevant gender‐based comparative analysis from the Native Women's Association of Canada, that address the impacts of colonization in research parameters, as well as the Ownership, Control, Access and Possession of Research Information (OCAP) Principles, and Participatory Action Research methods, that advocate for the meaningful participation of Aboriginal women in research. The guidelines also recognize the importance of using Indigenous languages, ensuring confidentiality, as well as giving something back to the community beyond financial compensation. They emphasize community permission, consultation, and consent, as well as including Aboriginal women in defining research priorities, choosing methodologies, analyzing and interpreting data, and disseminating and taking up knowledge. According to the report, the voices of Aboriginal women are essential to include in research because (1) they are “the keepers of specific knowledge (e.g., the use of medicinal plants, properties of berries, water management, crafts, etc.) and they need equal representation in all discussions and decisions that deal with the protection of that knowledge”; (2) their knowledge is “essential to a holistic vision of the world and of research”; and (3) including them will contribute to the decolonization of knowledge and the reappropriation of knowledge.^74^

These guidelines offer an alternative picture of research ethics to the one in the TCPS—a picture that recognizes the unique epistemic positions held by Aboriginal women with regard to particular forms of knowledge (e.g., knowledge related to plants and berries) and that promotes research that includes Aboriginal women as legitimate contributors and participants in research. Moreover, they resist hegemonic and colonial forms of knowing, in which the scientific method is the primary route to knowledge, and of recognizing knowledge, in which degrees and institutions are the basis of expertise. By offering alternatives to these assumptions, the guidelines introduce considerations that go beyond those covered in the TCPS, providing new touchpoints for Indigenous women, decision‐makers in Indigenous communities, and researchers, who may be developing or assessing a given research project.

There are more examples of guidelines for research that have been produced by communities who do not feel heard or represented by the TCPS. These include the *Principles of Ethical Métis Research*, *First Nations in Quebec and Labrador's Research Protocol*, and the *Protocols and Principles for Conducting Research in a Nuu‐Chah‐Nulth Context*.^75^ Non‐Indigenous communities, many of which include Indigenous members but do not self‐define through their indigeneity, have also produced research ethics guidelines such as *Ethical Research and Evidence‐Based Practice for Lesbians and Gay Men*, *Research 101: A Manifesto for Ethical Research in the Downtown Eastside*, and the *Principles for Conducting Research in the Jane Finch Community*.^76^

The documents referenced here are just some examples of significant and underutilized, if not entirely unrecognized, wisdom regarding research ethics. If these community‐led resistances are to shift the epistemological infrastructure undergirding Canada's system of research oversight, pathways of uptake need to be established.

### Training

Finally, resistance to the TCPS CORE training, and the assumptions that underlie it, can be found in community‐developed training programs. As a result of the difficulties of accessibility related to CORE experienced by several members of the OPPRA research team described above, they decided to develop Community Engaged Research Ethics Training. This training program offered workshops instead of the online training. The workshops took place in person, in a relaxed and interactive format, and covered both material from the TCPS and existing local guidelines. Those involved in OPPRA noted that the content of CORE did not reflect the ethical issues relevant to their project, including those issues related to community‐based participatory research, peer research assistants, or the unique context of the Downtown Eastside.^77^ As a result, they were able to incorporate local ethical issues and considerations into their novel research ethics training program. The Community Engaged Research Ethics Training initiative was an innovative and helpful response to some of the challenges posed by the federal training program. It also demonstrates how the current training opportunities serve to limit who is likely to be considered a researcher or arbiter of research ethics.^78^

Another recent example comes from the Jane and Finch community of Toronto that, in partnership with York University, established the Jane Finch Community Research Partnership (JFCRP), an initiative that aims to oversee all research occurring in the Jane and Finch community. As part of the community review process, researchers and students are required to complete the JFCRP research modules, a set of eight online training modules on research ethics with the community.^79^ These modules, along with the local guidelines, *Principles for Conducting Research in the Jane Finch Community*, foster a holistic understanding of the community's experiences with research, its research priorities, and its expectations of researcher conduct. According to the guidelines, “[t]he communities’ marginalized status has led to many professors and students working from deficit perspectives and operating from saviour complex standpoints, which has resulted in many researchers conducting research projects that aim to save the community from its many documented ills. In turn, this has resulted in many Jane and Finch residents often complaining of being ‘over‐researched’ and patronized as the community is treated as a research laboratory by York (and other institutions), and the effect is ultimately having their voices misrepresented.”^80^

The training modules are cocreated and facilitated by community members who bring their community‐based expertise to research and research ethics. The JFCRP training program is an accessible resource that provides researchers and community members with theoretical and practical knowledge to build scientific and ethical capacity for participating in community‐engaged research in the Jane and Finch neighborhood.

In addition to providing community‐led training to researchers, the JFCRP also has in place an advisory group, which is a group of community members responsible for reviewing and approving research protocols, providing feedback to researchers, and ensuring that researchers have successfully completed the Research Modules training program.^81^ The JFCRP advisory group ensures that community voice and community expertise are represented within research ethics review processes and provides ethical review in addition to the university‐based REB review. Taken together, this structure of research governance ensures that research is accountable to the Jane and Finch community at multiple levels. The existence of the training modules suggests to researchers that they must learn about the particularities of this community before attempting to conduct research there. Rather than merely applying universal principles or considerations from the TCPS, the JFCRP Research Modules establish a new epistemic hierarchy, one that places local expertise at the top.

While we have presented examples related to REBs, regulations, and training as distinct, it's essential to recognize the entanglement of these three components, and how the structures of knowledge promoted across them often serve to reinforce each other (e.g., training consists of learning about the TCPS, suggesting this is *the* relevant body of knowledge related to research ethics in Canada). This entanglement can also be seen in the process and procedures related to research ethics governance developed by communities. The JFCRP has developed a local review board, a set of local guidelines, as well as unique training modules, serving to disrupt and displace the entire system of research ethics developed federally. However, issues of authority and recognition remain; although researchers are encouraged to engage with the processes developed by the JFCRP, it is not required.

## CONCLUSION

Each of these examples highlights the rich forms of resistance that can be found alongside practices that maintain ignorance and produce injustice in research ethics governance. They also serve to reinforce the way in which marginalized communities and individuals often demonstrate epistemic advantages when it comes to knowledge projects that pertain to them. In a similar vein to feminist standpoint theory, Medina highlights three epistemic virtues that, he suggests, can be cultivated by those who are oppressed: *epistemic humility*, *epistemic curiosity/diligence*, and *epistemic open‐mindedness*.^82^ Together, Medina argues, these epistemic virtues converge in a kind of subversive lucidity that can enrich social cognition and reduce oppressive epistemic practices.^83^ As we have seen, such lucidity can be found in many communities across Canada that are resisting dominant portrayals of research ethics and offering alternative conceptions that reflect their local expertise. Whether they are being heard is less clear.

Medina offers these virtues in contrast to the vices of *epistemic arrogance*, *epistemic laziness*, and *epistemic close‐mindedness*, all of which may be more likely to turn up in those in power. In research ethics, these vices may be systematically supported, in that the epistemic picture of morality promoted in relation to research is one that is highly universalized and bureaucratized. Despite this, the forms of resistance highlighted above should give us hope for how we might counteract injustice and ignorance in Canadian research ethics. By attending closely to what resistant communities have to say, those in power, including researchers, REB members, and policy‐makers, can work to cultivate these epistemic virtues. As Medina puts it, in order to reduce the epistemic injustice that is woven into our lives and ways of relating, we need “disruptions, provocations, and, in short, resistance from others, so that our interactions with significantly different others can trigger productive self‐problematizations and beneficial epistemic friction.^84^ Such disruption, provocation, and resistance are clearly alive and well in community‐led forms of research governance across the country.

The analysis offered here calls for a re‐examination of the implicit epistemic assumptions built into the system of research ethics governance in Canada and a shift toward a more just system. Such a system would not only take up the wisdom of countless communities across the country that have worked to document their concerns and considerations but would also grant authority to those communities to speak on their own behalf, through processes of research review and/or through the publication of binding, local guidelines. It would also aim to teach researchers and research ethicists about more than the content of the TCPS, encouraging them to develop humility, curiosity, and open‐mindedness, and to listen to those who are speaking through resistance.

## ACKNOWLEDGMENT

This research was supported by the Social Sciences and Humanities Research Council (SSHRC) of Canada (grant # 430‐2020‐00327) as well as an internship funded in part by the Institute of Health and Social Policy at McGill University.
